# Effects of Iron Oxide Nanoparticles (Fe_3_O_4_) on Growth, Photosynthesis, Antioxidant Activity and Distribution of Mineral Elements in Wheat (*Triticum aestivum*) Plants

**DOI:** 10.3390/plants11141894

**Published:** 2022-07-21

**Authors:** Yingming Feng, Vladimir D. Kreslavski, Alexander N. Shmarev, Anatoli A. Ivanov, Sergey K. Zharmukhamedov, Anatoliy Kosobryukhov, Min Yu, Suleyman I. Allakhverdiev, Sergey Shabala

**Affiliations:** 1 International Research Centre for Environmental Membrane Biology, Department of Horticulture, Foshan University, Foshan 528000, China; fyingming@fosu.edu.cn (Y.F.); yumin0820@hotmail.com (M.Y.); 2 Institute of Basic Biological Problems, Russian Academy of Sciences, Institutskaya Street 2, Pushchino 142290, Russia; vkreslav@rambler.ru (V.D.K.); shurik_bx_04@mail.ru (A.N.S.); demfarm@mail.ru (A.A.I.); watcher01@rambler.ru (S.K.Z.); kosobr@rambler.ru (A.K.); 3 K.A. Timiryazev Institute of Plant Physiology, Russian Academy of Sciences, Botanicheskaya Street 35, Moscow 127276, Russia; 4 Tasmanian Institute of Agriculture, University of Tasmania, Hobart, TAS 7001, Australia; 5 School of Biological Science, University of Western Australia, Perth, WA 6009, Australia

**Keywords:** Fe_3_O_4_ nanoparticles, photosynthesis, respiration, phosphorous, iron, potassium, reactive oxygen species

## Abstract

Engineered nanoparticles (NPs) are considered potential agents for agriculture as fertilizers and growth enhancers. However, their action spectrum differs strongly, depending on the type of NP, its concentrations, and plant species per se, ranging from growth stimulation to toxicity. This work aimed to investigate effects of iron oxide (Fe_3_O_4_) NPs on growth, photosynthesis, respiration, antioxidant activity, and leaf mineral content of wheat plants. Wheat seeds were treated with NP for 3 h and plants were grown in the soil at two light intensities, 120 and 300 μmol (photons) m^−2^·s^−1^, followed by physiological assessment at several time points. High NP treatment (200 and 500 mg·L^−1^) enhanced plant growth, photosynthesis and respiration, as well as increasing the content of photosynthetic pigments in leaves. This effect depended on both the light intensity during plant growth and the age of the plants. Regardless of concentration and light intensity, an effect of NPs on the primary photochemical processes was not observed. Seed treatment with NP also led to increased activity of ascorbate peroxidase and reduced malondialdehyde (MDA) content in roots and leaves. Treatment with Fe_3_O_4_ also led to noticeable increases in the leaf Fe, P, and K content. It is concluded that iron oxide (Fe_3_O_4_)-based NP could enhance plant growth by improving photosynthetic performance and the availability of Fe and P.

## 1. Introduction

Applying innovative nanotechnology in agriculture is considered one of the promising approaches to obtain significant increases of crop yield [1,2]. There is a growing field of literature examining the effects of various types of nanoparticles (NPs) on plant growth and development [2,3,4]. Numerous reports show that NPs may increase crop production by enhancing different physiological processes including seed germination, photosynthetic activity, synthesis of various metabolites including proteins, and nitrogen-containing metabolites [3,5]. For example, CeO_2_ and TiO_2_ NPs had a positive effect on photosynthetic efficiency, mainly due to an increase in electron flow between photosystems II and I in the Hill reaction, as well as an increase in rubisco activity in the Calvin-Benson cycle [6,7,8].

It has been suggested that the positive effects of NPs on plants are associated with their large specific surface area, which leads to their high solubility and reactivity, and this determines their effective interaction with membranes and other cellular components, as well as with proteins and lipids [4,9,10].

In addition, nanotechnology increases the efficiency of fertilization, as treatment with NPs reduces soil pollution and the environmental risks of various chemical fertilizers [11]. At the same time, there are also significant reports of negative effects of NPs on plant performance. For example, application of nano-CuO inhibited seed germination and caused damage to root cells in rice [12]. Maize treated with TiO_2_ nanoparticles underwent rapid inhibition of leaf growth and transpiration [13], most likely due to its interference with Mg^2+^-ATPase operating in the chloroplast thylakoid membrane [6]. ZnO nanoparticles caused a reduction of biomass and cytological changes in the root cortical cells in *Lolium perenne* [14].

Different nanomaterials can be divided into four categories: macronutrient nanofertilizers, micronutrient nanofertilizers, nutrient-loaded nanofertilizers, and plant-growth-enhancing nanomaterials [1]. Among new nanoparticulates, TiO2 NPs and carbon nanotubes are considered plant growth enhancers although their mode of action remains obscure. Macronutrient nanofertilizers are chemically comprised of one or more macronutrient elements such as N, P, K, Mg, and Ca, while micronutrient nanofertilizers often contain Fe, Cu, Zn, Mn, and Mo NPs.

It appears that the action spectra of NPs differ strongly, depending on the type of NP, its concentrations, size, and the plant species affected. In this context, different effects on plants’ operations can be caused by NPs of the same type but different sizes or shapes. For example, small-sized CuO NP (25 nm) led to increased Cu uptake in seeds and significantly improved Cu content in soybean seeds, despite reduction in root size [15]; these effects were not reported for larger-sized CuO NPs (50 nm and 250 nm) nor Cu^2+^ ions. The same group of authors also reported differences in efficiency of different shaped ZnO NPs (spherical vs. floral-like vs. rod-like) on antioxidant defense systems and seed yield in soybeans [16]. Thus, the practical application of NPs as potential growth enhancers requires optimization of this technology on a case-by-case basis, as effects of NPs dependent on particle size, surface charge, and concentration may vary from beneficial to phytotoxic [17].

Colloidal solutions containing biologically active metals are now being widely used alongside traditional biological preparations [18]. The interaction of NPs with their surrounding molecules can be evaluated by monitoring the surface area and charge of the NPs. NPs can form aggregates in soil; however, plant root exudates, such as organic acids, enhance their dissolution [19]. Particle size and the extent of aggregation seem to have a direct correlation with the toxicity of NPs, with a decrease in particle size leading to increased stimulatory effect at low particle concentrations [20].

Analysis of data on absorption and translocation of Zn and Cu after treatment of wheat plants with CuO and ZnO NPs, and comparison these data with literature results, demonstrated that there are many pathways for the transformation and translocation of these NPs [19]. The dissolved Zn and Cu ions are taken up by plant roots and translocated into plant aerial portions to form complexes with different organic ligands. At the same time, and depending on the NP type, intact NPs can be absorbed and translocated into aerial portions via the xylem stream. Regarding iron oxide NPs, Zhu et al. demonstrated that a significant quantity of Fe_3_O_4_ NPs suspended in a liquid medium were absorbed by different pumpkin tissues, including leaves [21].

As plant growth and biomass gain are ultimately related to plants’ capacity for CO_2_ assimilation, several nanoparticles have been investigated for their potential beneficial effects on photosynthesis. The utilization of nanosilica (Nano) in photosystem II (PSII) increased the rate of photosynthesis in cotton plants [22], and Y (II) in rice increased by 10% to 17% after application of various doses of nano-TiO_2_ [23]. Concentrations of CeO_2_ NPs were effective for reducing the level of reactive oxygen species (ROS) and protecting chloroplasts, explaining the beneficial effects of CeO_2_ NP on alleviation of salinity-induced decline in PSII efficiency in some species [24]. The antioxidative effect of CeO_2_ NPs was also essential for minimizing negative effects of drought stress on photosynthesis and grain yield in sorghum [7].

Iron (Fe) is an essential micronutrient for plants and plays a key role in regulating plant growth and development including numerous cellular processes, such as chlorophyll biosynthesis, photosynthesis, chloroplast development, and dark respiration [25,26,27]. Iron also contributes to RNA synthesis and the Calvin cycle and is essential for the operation of certain respiratory enzymes [28]. A substantial proportion of iron in plants is localized in chloroplasts. Therefore, it is not surprising that iron deficiency causes changes in the structure and function of the entire photosynthetic apparatus of higher plants, leading to disturbances in the stoichiometry of photosystems and their lipid composition, and altering the a and b ratio of chlorophyll [29]. Fe is also essential for the activity of rubisco [28] and plays a role in stomatal closure [30,31]. However, the application of iron in ionic and chelated forms presents a number of issues compared to iron in the form of NPs.

Iron oxide and iron NPs are much smaller than typical iron oxide or iron molecules. They can create more complexes with different molecules and provide higher iron availability to plant organs [32,33]. In addition, similar to zinc and copper, iron NPs are typically absorbed more gradually while their ionic forms are taken up quickly and immediately included in various biochemical reactions [18].

Effects of iron oxide NPs were summarized in a review by Siddiqi and Husen [34]. However, this work hardly covered the mechanistic basis of the increase in various growth parameters, photosynthesis, and respiration; neither did they address the impact of light intensity and plant age.

Amongst different modes of NP application, seed priming is arguable the most attractive; other approaches such as leaf treatment or the addition of NPs into soil are more difficult to implement in practice. Upon seed priming, the seed passes through the metabolic and biochemical processes necessary for germination, which can stimulate the food quality and crop productivity. In addition, seed priming can positively affect the vigour of seedlings by activating the metabolic systems of plants, which is beneficial for seedling growth. The work of Sundaria et al. demonstrated that seed priming by iron oxide Fe_2_O_3_ NPs (size 80 nm) in two contrasting wheat genotypes induced germination, improved growth parameters (root and shoot length) [35] and Chl content, and enhanced accumulation of Fe in the grain, but the mechanistic basis of this process remains unknown. In addition, there is little data on nutrients and their effective translocation to different organs of plants treated by iron oxide NPs.

This work aimed to investigate the effects of iron oxide (Fe_3_O_4_) NPs on growth, photosynthesis, respiration, antioxidant activity, and mineral content distribution in wheat plants, in order to understand its mode of action and establish an optimal treatment regime.

## 2. Results

### 2.1. Growth

**Figure 1:**
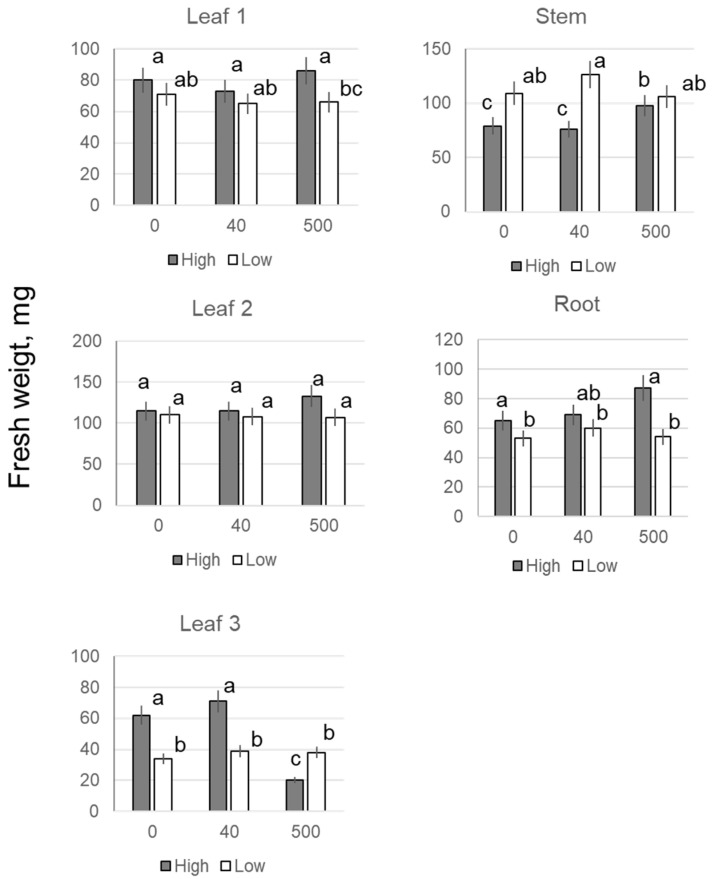
Effects of seed treatment with various concentrations of Fe_3_O_4_ nanoparticles on fresh weight of leaves, roots, and stems of wheat plants during their cultivation under high (300) and low light intensity (120 μmol (photons) m^−2^·s^−1^). Data are means ± SD (*n* = 5). Data labelled with different low-case letters are significantly different at *p* < 0.05.

The appearance of the third leaf began on day 13 of plant growth, and no difference was found at that point in the biomass allocation in the first, second or third leaves with any NP treatment up to 500 mg·L^−1^ (Table 1). Also, no difference was reported for plant roots (data not shown). In 19-day-old plants, no difference was found in the biomass of the first or second leaves at any concentration of NPs; however, the biomass of the third leaf after 200, 500, and 1000 mg·L^−1^ NP treatments was higher than that of the leaves of untreated control plants, and for 40 mg·L^−1^ NP treatment (Table 1). The biomass of roots increased by approximately 1.3 times in 200 and 500 mg·L^−1^ NP treatments. The aboveground plant biomass was significantly higher than the control only in the treatment with 500 mg·L^−11^ NP. Changes in dry weight were broadly in line with changes in fresh weight (FW). In 23-day-old plants, beneficial effects of NPs on biomass allocation were observed for 40, 200, and 500 mg·L^−1^ treatments, with the biomass of the fourth leaf being 7–10 times higher than the control (Table 1). The above effects were specific for iron nanoparticles and were not observed when Fe chelate was used as a mock control (Supplementary Figure S1). Cultivation in a weaker light (120 μmol quanta m^−2^·s^−1^) did not lead to any apparent difference between the control and the various concentrations of 40, 200 and 500 mg·L^−1^ of Fe_3_O_4,_ in terms of the biomass of roots or corresponding leaves (Figure 1).

### 2.2. Photosynthesis and Respiration

**Figure 2:**
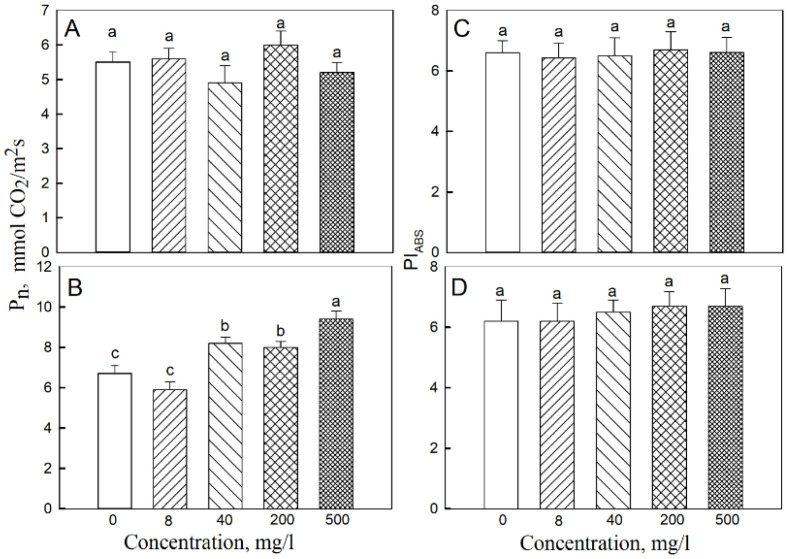
Effects of seed treatment with various concentrations of Fe_3_O_4_ nanoparticles on the rate of CO_2_ assimilation (P_n_) and PSII productivity index (PI_ABS_) in (**A**,**C**) second and (**B**,**D**) third leaves of 19-day-old wheat plants grown at a light intensity of 300 μmol (photons) m^−2^·s^−1^. Data are means ± SD (*n* = 5). Data labelled with different lower-case letters are significantly different at *p* < 0.05.

**Figure 3:**
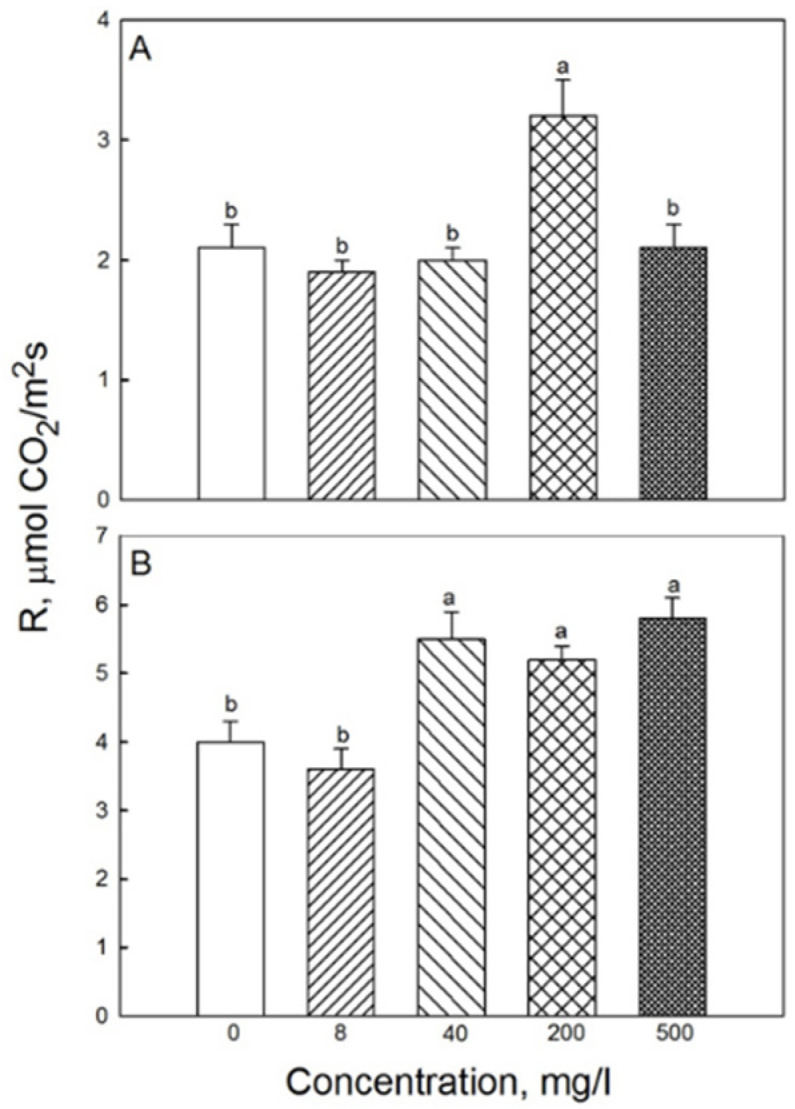
Effects of seed treatment with various concentrations of Fe_3_O_4_ nanoparticles on respiration (R) in (**A**) second and (**B**) third leaves of 19-day-old wheat plants grown at a light intensity of 300 μmol (photons) m^−2^·s^−1^. Data are means ± SD (*n* = 5). Data labelled with different lower-case letters are significantly different at *p* < 0.05.

The photosynthesis rates in the second and third leaves were determined. The highest rate of CO_2_ assimilation in the third leaf was observed when the concentration of iron nanoparticles was 500 mg·L^−1^, and the lowest was in the control and at 8 mg·L^−1^ of NPs (Figure 2). The respiration rate of the second leaf at 200 mg·L^−1^ of NPs was higher than in the other treatments, and treatments with 40, 200, and 500 mg·L^−1^ NP resulted in a higher respiration rate in the third leaf compared with the control (Figure 3). The respiration rate of the root system was similar for all treatments, ranging around 7.5–8 μmol CO_2_ m^−2^·s^−1^. The maximum carbon balance estimated as the (P_n_-R) difference was maximal when the seeds were treated with a 500 mg·L^−1^ of Fe_3_O_4_ NP. For the second leaf, there was no clear difference in these parameters between all treatments, except for the respiration rate at a 200 mg·L^−1^ concentration of NP. Stomatal conductance rates in third and second leaves were 0.25 ± 0.01 mol·m^–2^·s^−11^ and 0.30 ± 0.01 mol·m^–2^·s^−11^, respectively, and did not differ between controls and 500 mg·L^−1^ NP treatments. Also, no significant effects of NP treatment were reported for the transpirations rates in second or third leaves (3.76 ± 0.26 and 3.05 ± 0.21 mmol H_2_O, m^−2^·s^−1^, respectively).

**Figure 4:**
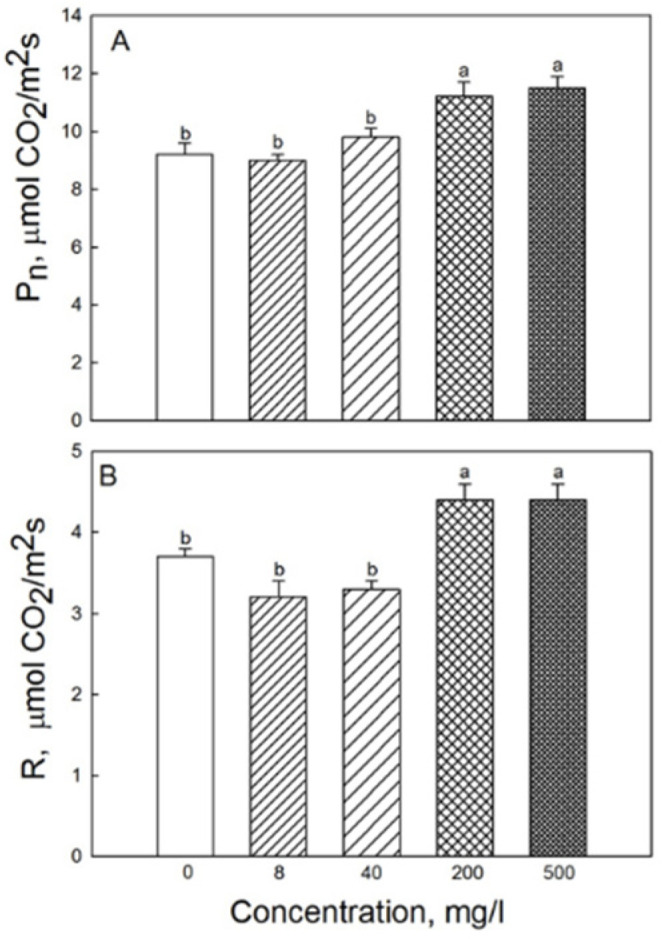
Effects of seed treatment with various concentrations of Fe_3_O_4_ nanoparticles on the rate of (**A**) CO_2_ assimilation and (**B**) respiration in third leaves of 19-day-old wheat plants grown at light intensity 120 µmol (photons) m^−2^·s^−1^. Data are means ± SD (*n* = 5). Data labelled with different lower-case letters are significantly different at *p* < 0.05.

In plants grown under low irradiance, P_n_ and R rates for 200 and 500 mg·L^−1^ NP treatments were significantly higher than in the control (Figure 4).

### 2.3. Photochemical Activity

**Figure 5:**
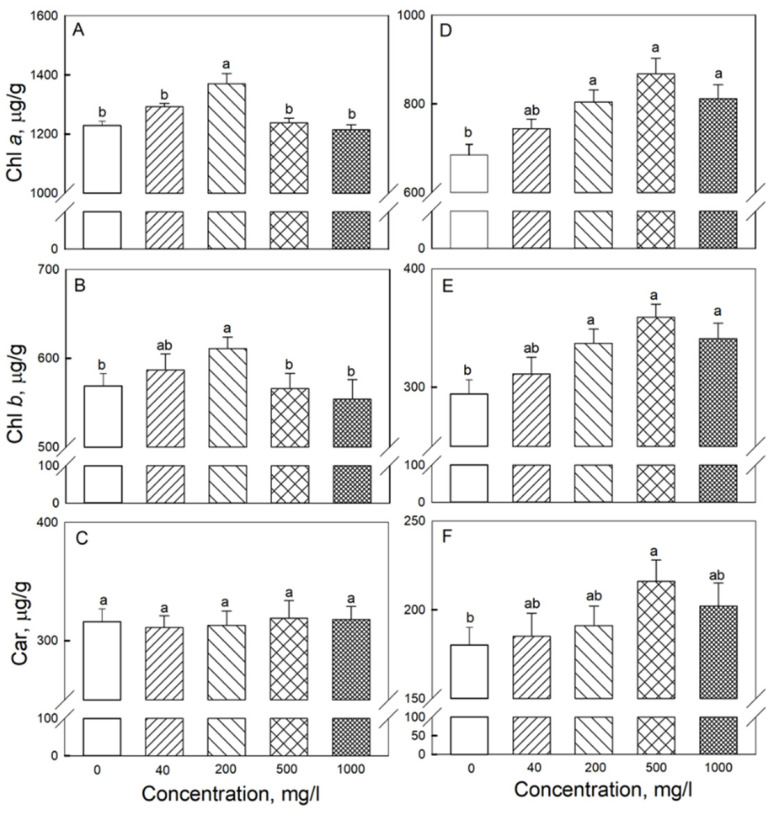
Effects of seed treatment with various concentrations of Fe_3_O_4_ nanoparticles on pigment content in (**A**–**C**) second and (**D**–**F**) third leaves of 19-day-old wheat plants grown at a light intensity of 300 μmol (photons) m^−2^·s^−1^. (**A**,**D**)—chlorophyll a; (**B**,**E**)—chlorophyll b; (**C**,**F**)—carotenoids (Car). Data are means ± SD (*n* = 5). Data labelled with different low-case letters are significantly different at *p* < 0.05.

The photochemical activity assessed by PI_ABS_ was practically the same for the third and second leaves when comparing any of the treatments with each other (shown for 19-day-old plants in Figure 2). With 200 mg·L^−1^ NP treatment, the content of Chl a, Chl b was higher in the second and third leaves than in the control; in the third leaf, beneficial effects of NPs on pigment content were observed for 200, 500, and 1000 mg·L^−1^ treatments (Figure 5).

### 2.4. Antioxidant Activity

**Figure 6:**
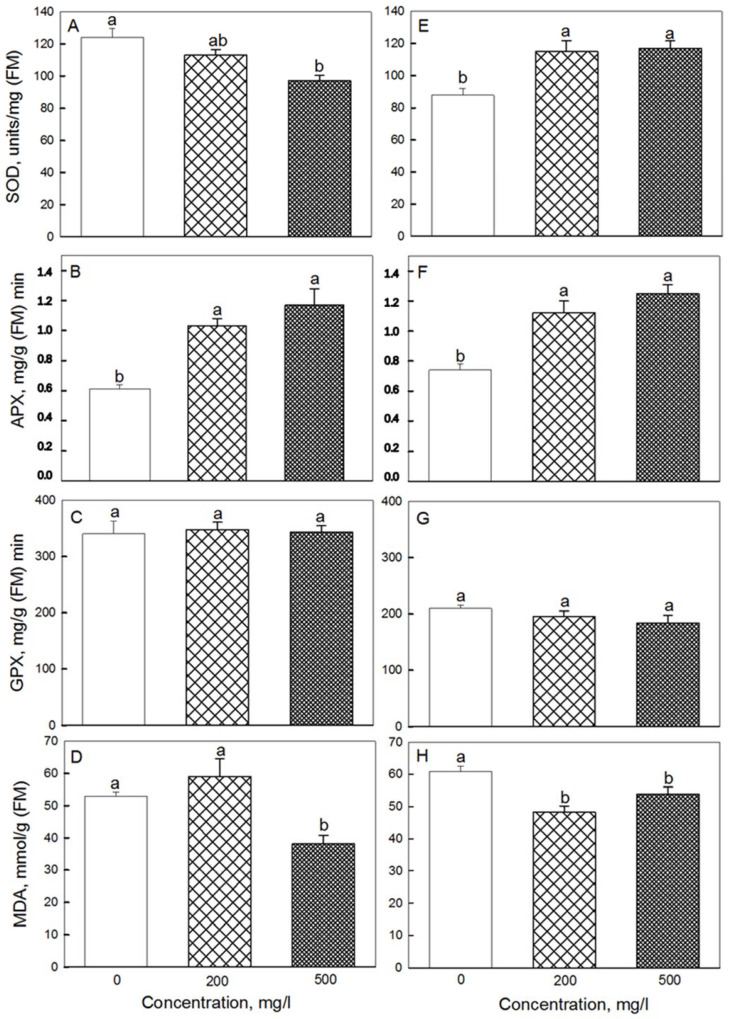
Effects of seed treatment with various concentrations of Fe_3_O_4_ nanoparticles on the activity of antioxidant enzymes: SOD (**A**,**E**) and APX (**B**,**F**), GPX (**C**,**G**), as well as MDA content (**D**,**H**) in (**A**–**D**) second and (**E**–**H**) third leaves of 19-day-old wheat plants grown at a light intensity of 300 μmol (photons) m^−2^·s^−1^. Data are means ± SD (*n* = 3). Data labelled with different lower-case letters are significantly different at *p* < 0.05.

A noticeable increase in APX activity compared to the control was observed for 200 and 500 mg·L^−1^ NP treatments in both the second and third leaves (Figure 6). Superoxide dismutase (SOD) activity was higher than control only in the third leaf. At the same time, no noticeable difference in glutathione peroxidase (GPX) activity was found between control and any of NPs treatments. The MDA content in the second leaf at 500 mg·L^−1^ of NPs was lower than in the control, and in the third leaf at 200 mg·L^−1^ of NPs. In roots, MDA content in plants treated with 500 mg·L^−1^ NP was 20% lower compared with controls (data not shown).

### 2.5. Elemental Content

**Figure 7:**
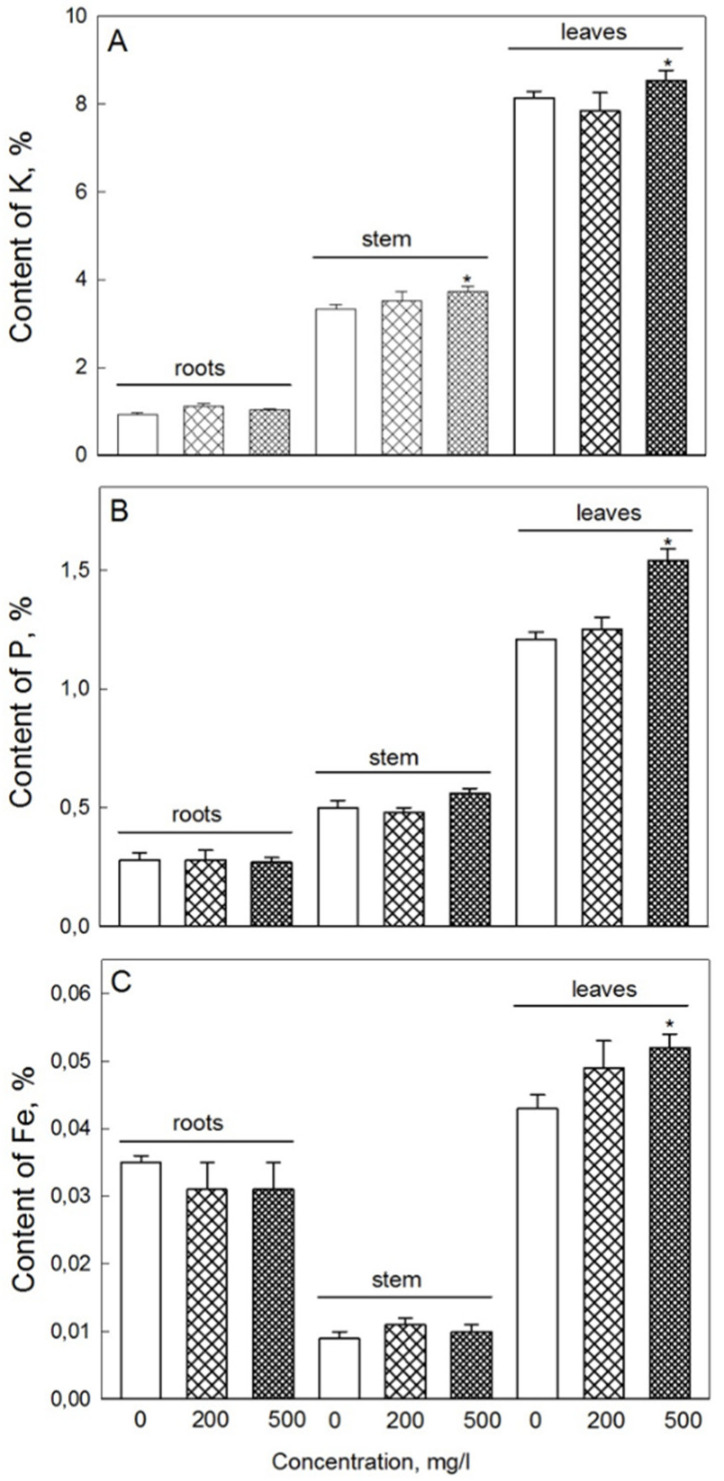
Elemental composition (amount of potassium (**A**), phosphorus (**B**), and iron (**C**) per dry weight basis) in roots, stems, and leaves of 19-day-old wheat plants treated with various concentrations of Fe_3_O_4_ NPs. Data are means ± SD (*n* = 4). Data labelled with asterisks is significantly different from appropriate controls at *p* < 0.05.

NP treatment had no significant impact on Mg, Ca, S, or Mn content in either leaves or roots (data not shown). The content of iron in the shoots (all leaves) in the 500 mg·L^−1^ NPs treatment group was 20% higher than in the control, and the phosphorus content was 27% higher (Figure 7). In the roots and stems, the difference in the content of these elements between control and NP treatments was not significant. Potassium content in the stem was higher at 500 mg·L^−1^ NP compared with control, but no such difference was reported for either roots or leaves.

## 3. Discussion

Iron in nanoform is a frequent component of micronutrient nanofertilizers and in plants it is often considered a double action micronutrient. While being an essential micronutrient controlling numerous physiological processes [36], in its ionic form Fe can also be involved in the formation of toxic ROS and thus be detrimental to plants (e.g., when Fe^2+^ interacts with H_2_O_2_ leading to formation of hydroxyl radicals via Fenton reaction) [37]. Its comparatively small particle size and ability to induce ROS play a key role in nanoparticles’ toxicity [2,38]. To reduce the potential toxicity, we used large Fe_3_O_4_ nanoparticles about 95 nm in size.

An increase in malondialdehyde (MDA), which is one of the major decomposition products of polyunsaturated fatty acids in cell biomembranes, is often used as an oxidative stress marker in physiological assays [39]. We did not find an increase in lipid peroxidation, assessed by MDA content, in 19-day-old plant leaves after seed treatment with iron nanoparticles. On the contrary, a decreased content of MDA was observed in second and thirds leaves concomitant with increased activity of APX and SOD (Figure 6). Also lower was the level of MDA in the roots of NP-treated plants. Hence, NPs under our conditions did not demonstrate any significant toxicity; on the contrary, they reduced oxidative damage in plants exposed to high light irradiation.

The translocation of nanoparticles of iron oxide is highly dependent on growth conditions, as well as the plant species per se. For example, Fe_3_O_4_ nanoparticles were found in roots and shoots of hydroponically-grown pumpkin (*Cucurbita mixta*) plants but only in roots of soil-grown plants [21]. Our data demonstrate that treatment with Fe_3_O_4_ NPs leads to an increase in the iron content in plant leaves, suggesting a more intense transfer of iron ions from the soil to leaves. This may potentially be explained by the better translocation of iron in the leaves after seed treatment with NPs. However, the use of Fe-chelate did not lead to positive effects of iron on growth and photosynthesis. It seems to us that seed treatment with Fe-chelate or F_3_O_4_ NPs leads eventually to accumulation of iron in leaves. Positive effects of seed priming with different concentrations of Fe NPs on Fe content were indicated not only in wheat shoots and roots but also in grains [40].

The positive effects of treatment with Fe_3_O_4_ NPs on K accumulation could be due to iron-dependent activation of NADPH oxidases, since the activity of these enzymes is essential for controlling intracellular K+ homeostasis via ROS-gated ion channels [41]. Additional experiments involving RBOH mutants are required to test this hypothesis directly.

Higher content of phosphorus in leaves after treatment with NPs can result in formation of Fe-phosphate species, similar to those observed with Zn and Cu [19]. This may be causally related to development of the third leaf, as the differences between treatments and control were mainly apparent in the third leaves (data not shown). In addition, the content of potassium in the stem was higher at 500 mg/l NPs than in stems of untreated (control) plants. It is plausible to suggest that higher content of potassium during NP treatment is required for the higher rate of nutrient flow to leaves, compared with control.

No difference in plant phenotype was observed until the appearance of the 3rd leaf (Table 1). Apparently, it takes time for the accumulation of iron ions to stimulate growth in the leaves, while in the roots the process of iron accumulation is faster. This is hardly surprising, as radial and long-distance ion transport are often uncoupled. At a later stage of development, at sufficiently high concentrations of iron oxide (200, 500, and 1000 mg·L^−1^), accelerated development of plants was observed, which was expressed in an increased biomass of the third or fourth leaf (Table 1). The acceleration of growth was apparently due to the increased rates of photosynthesis and respiration in the third leaf, and correlates with the increased consumption of carbon, which is consistent with the largest difference in Pn-R at 500 mg·L^−1^ of iron oxide compared to control. This is consistent with the fact that application of Fe-chelate did not lead to elevated rates of photosynthesis and respiration associated with enhanced growth of leaves and roots. Hence, in our case Fe_3_O_4_ NPs demonstrated an advantage compared with Fe-chelate.

Under the conditions of our experiment, we observed a sufficiently high content of Chl (a + b) − 1.8 mg per 1 g of FW, with F_v_/F_m_ values exceeding 0.80 (data not shown), which indicates the optimal functioning of the photosynthetic machinery [42]. However, at high concentrations of Fe_3_O_4_ NPs, the content of photosynthetic pigments increased, and so did the rate of CO_2_ assimilation in the second and third leaves. Several possible explanations can be brought forward. First, Fe_3_O_4_ can affect photosynthesis by regulating activity of rubisco [28], so one can envisage that such a scenario occurred under our experimental conditions. Second, plants treated with Fe_3_O_4_ NPs also possessed higher content of potassium and phosphorus in their leaves. The presence of these elements is important for the maintenance of activity of many enzymes, including enzymes of the Calvin cycle and dark respiration [37,43]. Finally, some SOD isoforms in plants are dependent on Fe for their activation, so elevated Fe content in plant leaves (Figure 7C) could be the reason for higher SOD activity in leaves (Figure 6E), minimizing detrimental effects of ROS produced in high-light-treated leaves, thus increasing chlorophyll content (Figure 5) and, as a result, net rate of CO_2_ assimilation.

While plants grown under weak light showed increased respiration rates with NP treatment, a difference in leaf growth parameters was not observed. This suggests that respiration alone is not responsible for enhancement of growth. Conversely, stimulation of root biomass was observed at high light intensity, most likely due to increased respiration in the roots of treated plants.

While previous papers reported negative effects of NPs (including Fe oxide) on plant performance, our data reported here showed a beneficial impact of such treatment. This is likely to be associated with the mode of F_3_O_4_ NP application (via seed priming) and the relatively large size of the NPs. This conclusion is supported by findings of Sundaria et al. [35]. Positive effects on plants’ Fe content after seed priming with different concentrations of Fe-based NPs were indicated not only in wheat shoots and roots, but also in grains [40]. However, the necessary quantities to increase the productivity of wheat at the final stage of plant development are unclear in each case. The final result depends on many factors, including wheat variety, type of soil, type of treatment, size, chemical properties of NPs, and environmental conditions [1,10,44].

## 4. Materials and Methods

### 4.1. Growth and Treatments Conditions

Wheat (*Triticum aestivum* L.) seeds of variety Moscowskaya 35 were obtained from Rusagro Group (Moscow, Russia). Seeds were sterilized in 5% hydrogen peroxide solution for 10 min followed by rinsing three times with deionized water.

Fe_3_O_4_ nanoparticles (99% purity; Advanced Powder Technology, Tomsk, Russia) were used in this study. The particle size was 80–110 nanometers, the bulk density 2.2 g/cm^3^, and BET specific surface area ~30 m^2^/g. Fe_3_O_4_ nanoparticles were prepared in double-distilled water and exposed to three hours of treatment by ultrasonic generator UZG13-0.1/22 (Ultrasound Technology, St. Petersburg, Russia) with a frequency of 22 kHz. The tested working concentrations of Fe_3_O_4_ ranged from 0.8 to 1000 mg·L^−1^.

Wheat seeds were treated with Fe_3_O_4_ solution by shaking in tubes for three hours by shaker (Rotamix RM-1; ELMI, Riga, Latvia). Seeds were then rinsed and put in Petri dishes with wet filter paper and placed in a thermostat at 23 ± 0.5 °C till for two days. As a mock control, seeds were treated with FeEDTA (Henan Honest Industrial Co., Ltd. Trading Company, Zhengzhou, China) solutions (20, 200 and 1000 mg/L) for three hours. The uniform seedlings were selected for planting and transferred to 1.2 L plastic pots filled with a potting mix containing a mixture of upper and lower peat, washed sand, lime meal (Garden Retail Service, Ivanteevka, Russia) with added 250 mg/kg nitrogen, 400 mg/kg phosphorus, 500 mg/kg potassium, and micronutrients (pH 6.5). Plants were grown for just over three weeks under white LED lamps at 24 ± 1 °C at two different irradiation levels: 300 and 110 μmol (photons) m^−2^·s^−1^ at 16 h photoperiod. Each pot contained 16 seedlings, and two pots were used for each treatment. Seedlings of uniform appearance were selected for physiological assessment on days 13, 19 and 23.

### 4.2. Agronomical Characteristics

The fresh and dry weight of first, second, third and fourth leaves and total shoot and root biomass were determined using an analytical balance (Scout Pro SPU123, Ohaus Corporation, Parsippany, NJ, USA) with an accuracy of 1 mg.

### 4.3. Pigment Contents

Determinations of the concentrations of chlorophyll a (Chl a) and b (Chl b) and total carotenoids (Car) in pigment extracts of all studied leaves were carried out spectrophotometrically by spectrophotometer (Genesis 10UV, ThermoSpectronic, Waltham, MA, USA) in 80% acetone as described elsewhere [45].

### 4.4. Gas Exchange Characteristics

The rate of CO_2_ assimilation (Pn), respiration (R) and transpiration (T) rates, and stomatal conductance (Gs) were determined in a closed system under light conditions using an LCPro + portable infrared gas analyzer from ADC BioScientific Ltd. (Hoddesdon, United Kingdom) connected to a leaf chamber with an area of 6.25 cm^2^. The CO_2_ uptake per leaf area (μmol m^−1^ 2s^−1^) was determined. The rate of photosynthesis in the second and third leaves of 19-day-old plants were determined at a saturating light intensity of 1000 μmol m^−2^·s^−1^. After measuring the rate of photosynthesis, the light was turned off, and the rate of dark respiration was measured.

### 4.5. Determination of Photochemical Activity

Fluorescence parameters characterizing the state of the photosynthetic apparatus were calculated on the basis of induction fluorescence curves obtained using data from the analysis of fast chlorophyll fluorescence induction kinetics in dark-adapted leaves (JIP-test), which is usually used to evaluate the state of PSII. Chl fluorescence induction curves (OJIP curves) were also recorded, according to the method described by Kreslavski et al. [46]. For the JIP test, OJIP curves were measured under illumination with blue light at an intensity of 5000 μmol (photons) m^−2^·s^−1^ for 1 s.

On the basis of induction fluorescence curves (OJIP curves), the following parameters that characterize the PSII photochemical activity were calculated: F_V_/F_M_, the PSII maximum quantum photochemical yield, and PI_ABS_, the PSII performance index [42,47]. Here, F_V_ is the variable fluorescence, which is equal to the difference between F_M_ and F_0_; F_0_ is the minimum amplitude of fluorescence (F), and F_M_ is the maximum amplitude of fluorescence. For calculation of the PI_ABS_, the following formula was used:

PI_ABS_ = (F_V_/F_M_)/(M_0_/V_J_) × (F_V_/F_0_) × (1 − V_J_)/V_J_) (1)

M_0_ = 4 × (F_300µs_ − F_0_)/(F_M_ − F_0_) (2)

V_J_ = (F_2ms_ − F_0_)/(F_M_ − F_0_) (3)

where M_0_ is the average value of the initial slope of the relative variable fluorescence of Chl a, which reflects the closing rate of the PSII reaction centers, and V_J_ is the relative level of fluorescence in phase J after 2 min.

### 4.6. Lipid Peroxidation

The extent of lipid peroxidation was evaluated by measuring the content of malondialdehyde (MDA), according to the method of Uchiyama and Mihara [48]. To obtain a plant extract, 0.25 g of leaves were ground in a mortar with 2 mL of cooled 100 mM K-phosphate buffer (pH 7.5) containing 0.1 mM Ethylene diamine tetraacetic acid (EDTA). The homogenate was centrifuged at 13,000× *g* for 15 min at 4 °C. The reaction mixture consisted of equal proportions (*v*/*v*) of the supernatant and a solution containing 0.8% thiobarbituric acid and 20% trichloroacetic acid. The mixture was heated in a boiling water bath for 15 min, then rapidly cooled on ice and centrifuged at 12,000 rpm for 5 min.

The MDA concentration of sample mixtures was measured at 532 and 600 nm by spectrophotometer (Genesis 10UV, ThermoSpectronic, Waltham, MA, USA). A mixture without plant extract was used as blank. The concentration of MDA was calculated using an extinction coefficient of 155 μmol^−1^ cm^−1^ and expressed as μmol/g (FM).

### 4.7. Plant Elemental Analysis

The contents of Ca, K, Mg, P, S, Mn, and Fe in various wheat tissues were determined by X-ray fluorescence (S6 Jaguar Spectrometer, Bruker, FRG, Billerica, MA, USA), according to the method described by Towett et al. [49].

### 4.8. Superoxide Dismutase Activity

SOD activity was determined by monitoring the inhibition of nitroblue tetrazolium (NBT) photochemical reduction at 560 nm, determined according to the method of Sen Gupta et al. with some modifications [50]. Blue formazin produced by NBT photoreduction was measured by the increase in absorbance at 560 nm. The reaction mixture without extract developed the most intense color and was used as a control. Complete reaction mixture without illumination was used as blank. A unit of enzymatic activity was defined as the amount of enzyme required to inhibit the rate of NBT reduction by 50% in comparison with a solution without enzyme.

### 4.9. Ascorbate Peroxidase Activity

The activity of ascorbate peroxidase (APX) was determined according to the method described by Veljovic-Jovanovic et al. with some modifications [51]. The APX activity was measured by monitoring the decrease in absorption at 290 nm (ε = 2.8 mM^−1^ cm^−1^) in a reaction mixture (2 mL) containing 50 mM K-phosphate (pH 7.2), 0.3 mM ascorbate, 0.1 mM H_2_O_2,_ and 100 μL of extract. A mixture without ascorbate was used as a reference solution. APX activity was then reported on a fresh weight basis.

### 4.10. Guaiacol-Dependent Peroxidase Activity

Guaiacol peroxidase (GPX) activity was determined according to Chance and Maehly’s method with some modifications [52]. The enzyme activity was measured by monitoring the increase in absorption at 470 nm (ε = 26.6 mM^−1^·cm^−1^) against a blank without the enzyme. GPX activity was then reported on a fresh weight basis.

### 4.11. Statistics

For chlorophyll fluorescence measurements, 6 to 10 leaves from at least four individual plants were used; similar numbers were used for enzymatic activity, pigment content, and gene expression data. In growth experiments, 16 plants were used for each treatment. The data shown in the tables and figures are mean ± SDs, where *n* represents a pooled sample composed from several biological replicates. The significance of differences between two data groups was determined by Student’s t-test at the 5% significance level. Comparison of the data between many groups was performed by a one-factor analysis of variance (ANOVA) and Tukey’s multiple comparison test. The variance homogeneity was confirmed by Levene’s test. For data processing and statistical analysis, we used R 3.5.0 (R Foundation for Statistical Computing, Vienna, Austria, 2017).

## 5. Conclusions

Seed priming with Fe_3_O_4_ NPs did not result in any toxic effects, as evident by lowered content of MDA and PSII activity (PI_ABS_) similar to control plants. Leaf photosynthesis was enhanced via mechanisms not associated with an increase in the activity of the primary processes of photosynthesis, nor increased stomatal conductance, but rather with the enhanced function of enzymes involved in the Calvin cycle. In addition, seed priming with iron oxide NPs increased leaf Fe and P content. Enhanced photosynthesis and elevated contents of these essential elements resulted in the overall enhancement in leaf growth.

## Figures and Tables

**Table 1:** Effect of seed treatment with various concentration of Fe_3_O_4_ nanoparticles on the fresh weight of various organs of wheat plants during their cultivation under high (300 μmol (photons) m^−2^·s^−1^) light intensity. SL − stem + leaves. Means ± SD (*n* = 5).

Seedling Age	[Fe_3_O_4_], mg·L^−^^1^	Biomass, mg						
		1st Leaf	2nd Leaf	3rd Leaf	Stem	SL	Roots	4th Leaf
13-d-old	0	75 ± 6	99 ± 10	4.3 ± 2.1	69 ± 6	248 ± 23	-	-
	40	71 ± 8	101 ± 9	3.7 ± 1.8	65 ± 8	241 ± 22	-	-
	200	77 ± 7	98 ± 11	3.1 ± 1.5	62 ± 7	220 ± 20	-	-
	500	83 ± 6	91 ± 8	8.3 ± 2.4	72 ± 8	254 ± 19	-	-
19-d-old	0	80 ± 7	115 ± 10	62 ± 7c	79 ± 6	338 ± 28	66 ± 5	-
	40	73 ± 4	115 ± 10	71 ± 8bc	76 ± 6	337 ± 31	69 ± 7	-
	200	79 ± 5	129 ± 8	87 ± 6 *	94 ± 11 *	388 ± 30	85 ± 6 *	-
	500	86 ± 6	133 ± 15	120 ± 11 **	98 ± 10 *	437 ± 35 *	87 ± 7 *	-
	1000	87 ± 7	130 ± 12	122 ± 9 **	96 ± 7 *	441 ± 33 *	88 ± 8 *	-
23-d-old	0	71 ± 5	97 ± 10	148 ± 13	92 ± 8	411 ± 35	79 ± 6	3 ± 1
	40	69 ± 4	94 ± 8	156 ± 17	89 ± 7	434 ± 38	80 ± 7	26 ± 3
	200	73 ± 7	105 ±11	174 ± 15	95 ± 8	467 ± 44	93 ± 5	20 ± 3
	500	71 ± 6	114 ± 9	168 ± 12	96 ± 6	482 ± 37	98 ± 6 *	32 ± 7

* and ** indicate significant differences between experiment and control (no Fe_3_O_4_) at *p* < 0.05 and *p* < 0.01, respectively, by the Student *t*-test.

## Data Availability

Not applicable.

## Supplementary Materials

The following supporting information can be downloaded at: https://www.mdpi.com/article/10.3390/plants11141894/s1, Supplementary Figure S1: Effects of seed treatment with different concentrations of iron chelate (FeEDTA) on leaf and root fresh weight and rates of photosynthesis and respiration, in 19-d-old wheat plants during cultivation under light intensity of 300 μmol (photons) m^−2^·s^−1^. Means ± SD (*n* = 3).
