# Supplementary material for: Effects of Iron Oxide Nanoparticles (Fe3O4) on Growth, Photosynthesis, Antioxidant Activity and Distribution of Mineral Elements in Wheat (Triticum aestivum) Plants
Source: Plants (Basel). 2022 Jul 21;11(14):1894. doi: 10.3390/plants11141894 (PMC9322615; doi:10.3390/plants11141894)
Supplement: Supplementary file 1 [file plants-11-01894-s001.zip › plants-1803889-supplementary.pdf]

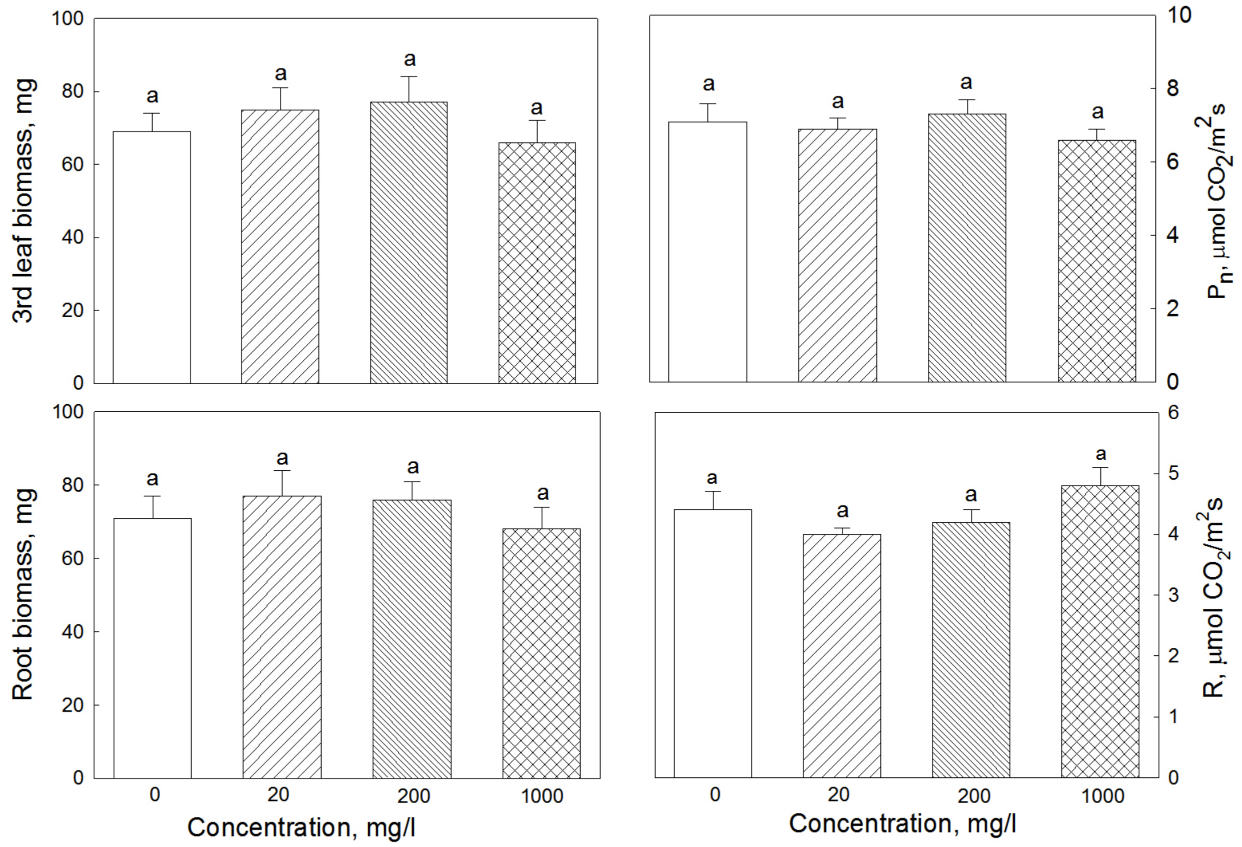

Supplementary Figure S1. Effect of seed treatment with different concentrations of iron chelate (FeEDTA) on leaf and root fresh weight and rate of photosynthesis and respiration of 19-d-old wheat plants during their cultivation under light intensity of 300 μmol (photons) m^{-2} s^{-1}. Means ± SD (n =3).
